# Supplementary material for: Chinese herbal medicine for children with idiopathic short stature (ISS): A systematic review and meta-analysis
Source: PLoS One. 2022 Jun 24;17(6):e0270511. doi: 10.1371/journal.pone.0270511 (PMC9232169; doi:10.1371/journal.pone.0270511)
Supplement: Attachment — Submitted filename: Rebuttal Letter.doc [file pone.0270511.s002.doc]

1^st^ of June 2022

Dear reviewers:

Thank you for your specific and helpful comments. The manuscript has been improved according to the suggestions of the reviewer:

Major points

1. As suggested by the reviewer, we have added the names of the database in the abstract.

2. The details of the software have been mentioned in the abstract and statistical analysis part.

3. We have added the details of the RCT types (study design section) in Table 1.

4. We have changed the sequence in the Methods section as per your suggestions.

5. The mean age, Number of participants, Number of Males/Females and mean Study duration were mentioned in Table 1. And we have also added a summary of these aspects of the included literature in the Research characteristics part.

6. We added the p-value in all the results that were able to be supported by a p-value.

7. The discussion of the strengths of the current findings of this paper is shown on lines 272 to 275.

Yours sincerely,

On behalf of the authors,

Jinghua Yang, MD
